# Cavernous hemangiomas of the temporalis muscle with prominent formation of phleboliths: Case report and review of the literature

**DOI:** 10.1097/MD.0000000000008948

**Published:** 2017-12-01

**Authors:** Bin Cui, Dan-Hui Wang, Guan-Jun Wang, Peng Cheng, Feng Zhang, Xiao-Bing Duan, Zhong-Fu Zhao

**Affiliations:** a Department of neurosurgery; b Department of neurology, Xuchang Central Hospital, Henan, China.

**Keywords:** hemangioma, phleboliths, temporalis muscle

## Abstract

**Rationale:**

Hemangiomas are benign tumors characterized by an abnormal proliferation of blood vessels, most often occur in the skin and subcutaneous tissue, intramuscular hemangioma, a distinctive type of hemangioma within the skeletal muscle, account for <1% of all hemangiomas, temporalis muscle is a very uncommon site, cavernous hemangioma of the temporalis muscle with prominent formation of phleboliths is rare reported.

**Patient concerns:**

A 62-year-old man presented with a slowly increased mass in his right temporal fossa.

**Diagnoses:**

Computed tomography (CT) scan showed the lesion across the zygomatic arch, with many calcified nodules differ in sizes and no erosion to the bone, magnetic resonance imaging (MRI) showed an oval lesion with hypointense and isointense on T2-weighted imaging within the temporal muscle, and preoperation diagnosis was hemangioma.

**Interventions:**

The tumor was resected under general anesthesia.

**Outcomes:**

The mass was excised completely, and the histopathology examination confirmed the diagnosis of cavernous hemangioma with prominent formation of phleboliths. The patient recovered very well without dysfunctions.

**Lessons:**

Cavernous hemangioma should be suspected when mass occurs in this region. CT and MRI are important for the early diagnosis of tumor, and resection the tumor completely is recommended.

## 1 Introduction

Hemangiomas are benign vascular neoplasms most often in infancy and childhood, and commonly involve skin and subcutaneous tissue, it is about 0.8% of all hemangiomas are intramuscular, with the trunk and extremities are frequently located. Only 14% of intramuscular hemangiomas are located in head and neck region, with the master, trapezius are the common sites, the temporalis muscle is an very uncommon site, and 90% intramuscular hemangiomas affected young adults and no sex performance are noted.^[1]^ According to Scott, hemangiomas arise in abnormal embryonic sequestrations that retain their embryonic characteristic, minor trauma, excessive muscle contraction, or the fluctuation of hormone is believed to play important factors in the growth of the mass by stimulating blood flow in the preexisting tumors.^[2,3]^

The clinical symptoms of intramuscular hemangiomas usually manifested as painless, slow growth, clear boundaries of the tumor, and the formation of phleboliths are calcified thrombi is one of the characteristics of hemangioma, occurring in 15% to 25% of intramuscular hemangioma, they are variable in size and number, and cause no subjective symptoms.^[4]^ Preoperative diagnosis of intramuscular hemangioma is difficult because of low incidence and lack of specific symptoms, >90% of cases are misdiagnosed before surgery.^[5]^ Computed tomography (CT), magnetic resonance imaging (MRI), and ultrasound examinations are essential for the diagnosis of intramuscular hemangioma. An additional case of cavernous hemangioma located in the temporalis muscle with multiple phleboliths here is reported.

## 2 Case report

**Figure 1:**
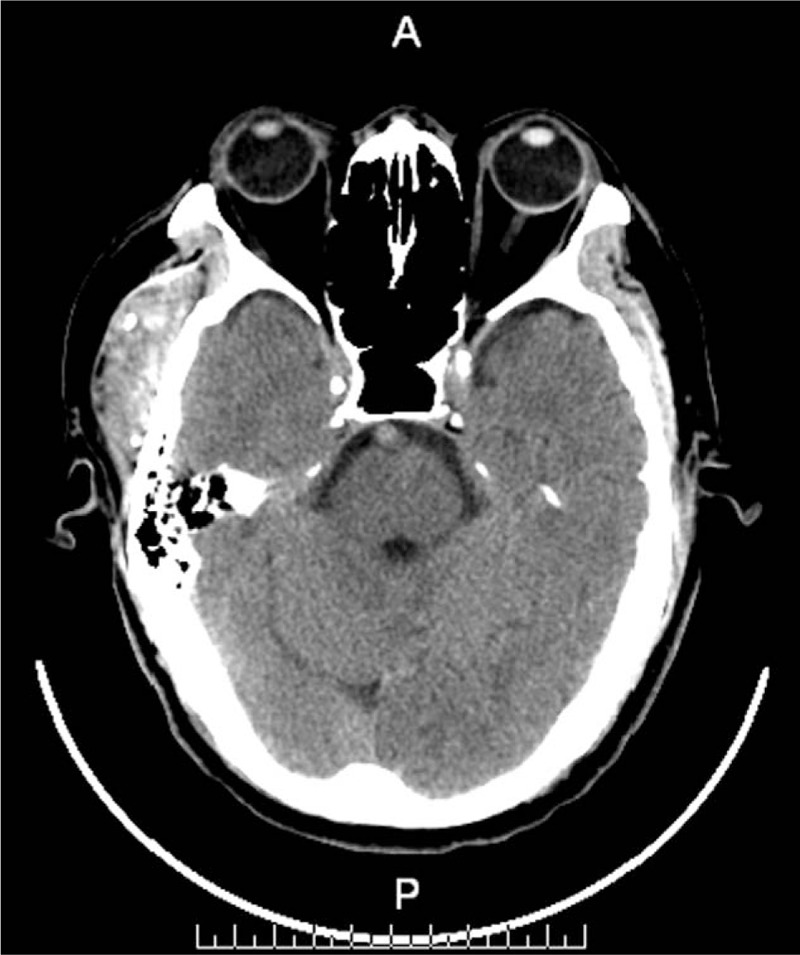
Computed tomography shows the mass in the right temporal region with many calcified nodules and no erosion of the bone.

**Figure 2:**
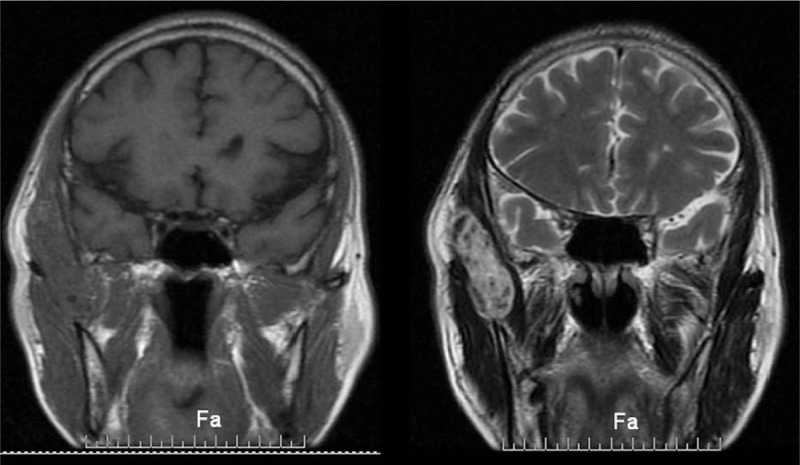
(A) T1-weighted coronal MRI scan shows a 6.2-cm ovoid mass within right temporal muscle and across the zygomatic arch. (B) T2-weighted coronal MRI scan shows millimetric hypointense structures in the mass. MRI = magnetic resonance imaging.

A 62-year-old man was admitted in our department, with a history of a mass in the right temporal fossa which had been gradually increasing in size over the last 3 years. Physical examination showed the size of the mass was about 3 × 2 cm in diameter, with painless, no pulsation and the overlying skin was normal. No history of trauma and surgery. Subsequently, CT scan showed the boundaries of the mass were clear, which with many calcified nodules differ in sizes and no erosion of the bone (Fig. 1). MRI scan was performed, which revealed a 2.2 × 5.1 × 6.2-cm ovoid mass within the right temporal muscle, across the zygomatic arch. The tumor had isointense signals on T1-weighted sequences and hyperintense signals on T2-weighted sequences, containing fields in which no signal septations or calcific foci were detected (Fig. 2).

**Figure 3:**
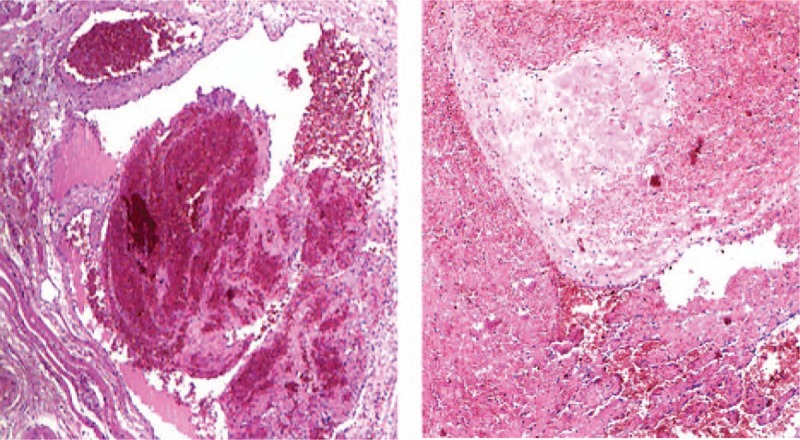
Histopathologic image shows cavernous vascular structures divided by fibrous septa. In part of vascular channels, we can see thrombi, and muscle tissue around it is visible.

The patient was taken operation under general anesthesia through a hemi-coronal flap, the skin incision was placed behind the hairline, a dark red soft tumor lying in the temporal muscle was encountered, many calcified nodules were found in the mass, and the mass was totally excision with a margin of normal muscle. Histopathological examination showed the mass composed of large cavernous vascular structures which divided by fibrous septa, thrombi were seen in part of vascular channels, and we found muscle tissue around it. The diagnosis was cavernous hemangioma with formation of phleboliths (Fig. 3). The patient recovered very well, 6 months after the operation, no sign of local recurrence was observed.

This case report was approved by the Ethics Committee of Xuchang Central Hospital (board, s name: Feng Niu).

## 3 Discussion

Hemangioma is a kind of vascular malformations, it is about 0.8% of all hemangiomas are intramuscular, with the trunk and extremities are frequently located. Only 14% are located in head and neck region, with the master (36%), trapezius (24%) are the common sites, the temporalis muscle is an very uncommon site. In 1843, intramuscular hemangiomas were first reported by Liston, and classified by Allen and Enzinger in 1972, depending on the vessel size. Intramuscular hemangiomas are classified into capillary hemangioma, cavernous hemangioma, and venous hemangioma.^[6]^ Capillary hemangioma is composed of small capillary-sized vessels, represents 68% of all intramuscular hemangiomas, followed by cavernous hemangioma which consists of thin-walled and cystic blood spaces, and the third is venous hemangioma with dilated veins with walls of varying thickness within loose fatty-fibrous stromas, with an incidence of 26% and 6%, respectively.^[7]^ Phlebolith formation within intramuscular hemangioma is present in 15% to 25% approximately, phleboliths consist of a mixture of calcium carbonate and calcium phosphate salts, the pathogenesis of phleboliths is thought to involve thrombi produced by slowing of peripheral blood flow, and then it becoming organized and mineralized, initially, calcified thrombi occurs, forming the core of the phlebolith. The fibrous component attaches to a developing phlebolith and becomes calcified. Repetition of this process causes enlargement of the phlebolith.^[4,8,9]^ It is regrettable that we have not done histopathological examination of the phlebolith.

The etiology of these lesions is unknown, although repeated trauma or hormonal changes cause growth of the tumor by resulting in the proliferation embryonic vascular tissue.^[2]^ A congenital theory has been proposed because of the high incidence of hemangiomas during the first 3 decades of life.^[10]^ Intramuscular hemangiomas with the formation of phlebolith usually present as a slow growing, painless mass before cause any symptoms, and do not exhibit any of the vascular signs or representative symptoms.^[11,12]^ When the tumor involved in the temporal fossa, the old man found it, maybe on account of slow growth of the tumor and deep location. The differential diagnosis includes neurofibroma, lipoma, dermoid cyst, enlarged lymph nodes, soft-tissue sarcoma, myositis ossificans and temporal arteritis, and so on.^[13]^ Because of low incidence and lack of specific symptoms, it is difficult to diagnose, and the accuracy of preoperative diagnose <8%,^[5,14]^ CT, MRI, and ultrasound examinations play an important role in the diagnosis of intramuscular hemangioma.

CT scan plays an important role in the identification the size and shape of the tumor and the surrounding tissues. We can see calcification and no erosion of the bone from the preoperative CT from this patient. Ultrasound on the diagnosis of masseter hemangioma is reliable, and for other types of hemangiomas can be used as an option.^[15]^ MRI is very important for the identification of tumor, the hemangioma had isointense signals on T1-weighted sequences and high signals on T2-weighted sequences, it is different from the surrounding tissues,^[16]^ and the phlebolith shows hypointense signal on T1/T2-weighted imaging. Certain radiologic features, on MRI, would appear to suggest hemangiomas: high-intensity signal on T2-weighted imaging; endothelial-lined vascular channels separated by fibrous and/or fatty linear tissue in lesions bigger than 2 cm; and presence of areas of thrombus, fibrosis, hemosiderin deposition, and/or calcification.^[16]^

The way to treat intramuscular hemangiomas include simple observation, irradiation, injection of sclerosing agents, corticosteroid treatment, embolization, and surgical excision. The treatment options should be individualized with respect to localization and depth of invasion. According to report, only 1 venous hemangioma in the temporal muscle disappeared spontaneously.^[17]^ The majority of hemangiomas involving the temporal muscle are cavernous hemangiomas, complete resection of the tumor is the ideal treatment, due to the invasive characteristics of hemangioma, surgical resection of a rim of normal surrounding tissue is conducive to the diagnosis of tumor classification and reduce the recurrence rate.^[10]^ Surgical indications include age, repeated hemorrhagic episodes, location and size of tumor, invasion depth, growth rate, refractory pain, cosmetic malformations, and suspected malignant transformation. For huge tumors, combined with injection of sclerosing agents and corticosteroid treatment is essential.^[18]^ Irradiation is not recommended for children because of the high dose of radiation and serious potential complications.^[19]^ The recurrence rates for the capillary hemangioma, cavernous hemangioma, and venous hemangioma were 20%, 9%, and 28%, respectively. Incompletely excision, invasive of the surrounding tissue can lead to increased recurrence rate.^[20]^ Due to the invasive biological characteristics (high recurrence rate, invasive growth), Heckl et al^[21]^ advocated that surgery is only applicable to capillary hemangioma, and at least, when presence of intractable pain, esthetic problems, and functional impairment, and with rigorous clinical observation and radiological follow-up at least 2 years.

The patient was taken tumor resection under general anesthesia; the skin incision was located behind the hairline, so that the cosmetic results will be excellent. Carefully separate the surrounding tissue and protect the facial nerve branch intraoperative, the amount of blood loss is about 50 mL. Subcutaneous effusion is serious due to the size of tumor, and the effusion completely disappeared, through the puncture and local pressure bandage. There was no recurrence of our patient 6 months after surgery.

## 4 Conclusion

Combined with radiologic findings, cavernous hemangiomas should be suspected when mass occurs in the temporal region. Due to the destruction of the surrounding tissue, cavernous hemangiomas need treatment generally. It is recommended to resection completely with a rim of surrounding muscular tissue for accessible tumor, without any serious dysfunctions.
